# Measurement of Three-Dimensional Back Shape of Normal Adults Using a Novel Three-Dimensional Imaging Mobile Surface Topography System (MSTS): An Intra- and Inter-Rater Reliability Study

**DOI:** 10.3390/healthcare11233099

**Published:** 2023-12-04

**Authors:** Gok Kandasamy, Josette Bettany-Saltikov, Paul Van Schaik

**Affiliations:** 1 School of Health and Life Sciences, Teesside University, Middlesbrough TS1 3BA, UK; j.b.saltikov@tees.ac.uk; 2 Centre for Applied Psychological Science, School of Social Sciences and Law, Teesside University, Middlesbrough TS1 3BA, UK; p.van-schaik@tees.ac.uk

**Keywords:** spinal deformity, musculoskeletal (MSK) disorders, three-dimensional imaging, spinal pain, posture screening, mobile surface topography

## Abstract

Postural and spinal deformities are major contributing factors to musculoskeletal (MSK) disorders. Posture screening and assessment can help to identify early morphological deformities, thereby preventing progression and reducing or correcting them with effective treatments. The study evaluates both intra- and inter-repeatability of using a mobile structured light sensor with a structured light pattern for building an accurate 3D human model and its use in postural screening. 16 young males (age: 25 ± 5.6 years, height: 172 ± 5.3 cm, mass: 69 ± 8.6 kg) participated without any musculoskeletal pain or pre-existing leg or spinal abnormalities. An iPad-based 3D mobile scanning tool, Structure Sensor^TM^ (2018 version), was used to capture the participants’ back and whole-body shape. The collected data (3D model) were realigned and processed in the open-source software, Netfabb Basic^TM^ (7.2 version). For each participant, five trained raters individually measured three trials of standing back and body posture on two separate occasions to calculate both intra- and inter-rater reliability. With the use of this software, nine postural variables and angular displacements were individually measured by the raters. The results indicated good to excellent intra-rater and good to moderate inter-rater reliability for measuring 78% (7 out of 9) of postural variables with an ICC ranging from 0.70 to 0.98. The remaining 22% of variables (2 out of 9; lateral pelvic tilt and right frontal knee angle) showed moderate to low inter- and intra-rater reliability, with ICCs ranging from 0.26 to 0.79.

## 1. Introduction

Musculoskeletal (MSK) disorders are the second most common cause of disability worldwide, with spinal pain being one the most frequently cited symptoms [1,2]. Anderson et al. [3] projected the worldwide annual incidence of spinal pain in adults to be 15%, together with a prevalence as high as 30%. One major contributing factor is postural or spinal deformity [4], as Significant asymmetries in back posture/deformity can lead to abnormal stress and loading on the spinal MSK structures [5].

Willner and Udén [6] and Yawn [7] suggest that early detection/screening is the most-effective management of spinal deformities. Recently, Prowse et al. [8] identified screening as a powerful tool to identify children who might perhaps have scoliosis and are at a high risk of developing it. Furthermore, Grivas et al. and Prowse et al. [8,9] suggest that the tools used for screening need to be easy to administer, portable, safe, and inexpensive, with the ability to provide essential topographical back surface information, thus replacing the need for repeated radiation from radiographs.

In posture screening and assessment, recent technological advancements, together with their application in biomechanics, have enabled a new mode of data capture. The key postural variables commonly measured in posture screening are lumbar lordosis, thoracic kyphosis, and external neck curvature in the sagittal plane, shoulder elevation and lateral pelvic tilt in the frontal plane, and scapular prominence in the transverse plane [10]. Capturing the 3D surface pattern using the Kinect sensor, together with its clinical implications, is one example [11]. This study evaluates both intra- and inter-reliability of using a mobile structured light sensor with a structured light pattern for building an accurate 3D human model, together with its use in postural screening.

The main aim of this study is to introduce a novel mobile surface topography system (MSTS) and evaluate the intra- and inter-rater reliability, for assessing back and full-body shape in normal subjects. The research question guiding this study is as follows: what is the extent of variation in intra-rater reliability and inter-reliability of the posture and back shape measurement variables using MSTS?

## 2. Materials and Methods

### 2.1. Study Design

The current experimental study was designed to investigate the intra- and inter-rater reliability of data outputs from a Novel Three-Dimensional Imaging Mobile Surface Topography System (MSTS) for standing posture and back shape. A convenience sample of 16 young males (age (SD): 25 (5.6) years, height (SD): 172 (5.3) cm, mass (SD): 69 (8.6) kg) participated in this study (Table 1). The participants were healthy asymptomatic subjects without any musculoskeletal pain or pre-existing leg or spinal abnormalities. Five raters (four males and one female) took part. The mean clinical experience of the raters was 10 (7) years for the two physiotherapists, 8.5 (4.9) years for the sports therapists, and 2 years for the biomechanist.

### 2.2. Materials

A commercially available iPad-based 3D mobile scanning tool, Structure Sensor^TM^, was used to capture the participants’ back and whole-body shape. The Structure Sensor is a 3D sensing accessory that uses an eye-safe infrared laser to project invisible structured infrared light onto an object and captures it with an infrared camera to produce a depth map (Structure Sensor, 2016). RGB-D cameras consist of an infrared (IR) projector, which emits a known pattern of structured IR light, an IR camera, and an RGB camera.

A 3D point is computed by relating and combining all decoded captured pixels and points in both rows and columns (x and y points), achieved by creating a mesh of an object through the triangulation method. The depth camera sensor is able to sense a depth of up to 12 feet; hence, it is highly suitable for scanning indoor rooms [12]. The Structure Sensor^TM^ captures 3D high-quality imagery data instantaneously, which helps create highly reliable 3D models with high resolution in seconds.

This sensor, along with the normal iPad camera, provides real-time anatomical landmarks and reconstructs the whole back and body shape using the triangulation method [13]. In the current study, the collected data (3D model) were realigned and processed in the open-source software, Netfabb Basic^TM^ (Version 7.2). This software was originally designed for 3D model building and printing in the fashion and civil engineering industries, as well as the mechanical industry. This tool has been adapted for use in the assessment of posture and back shape.

### 2.3. Method

**Figure 1:**
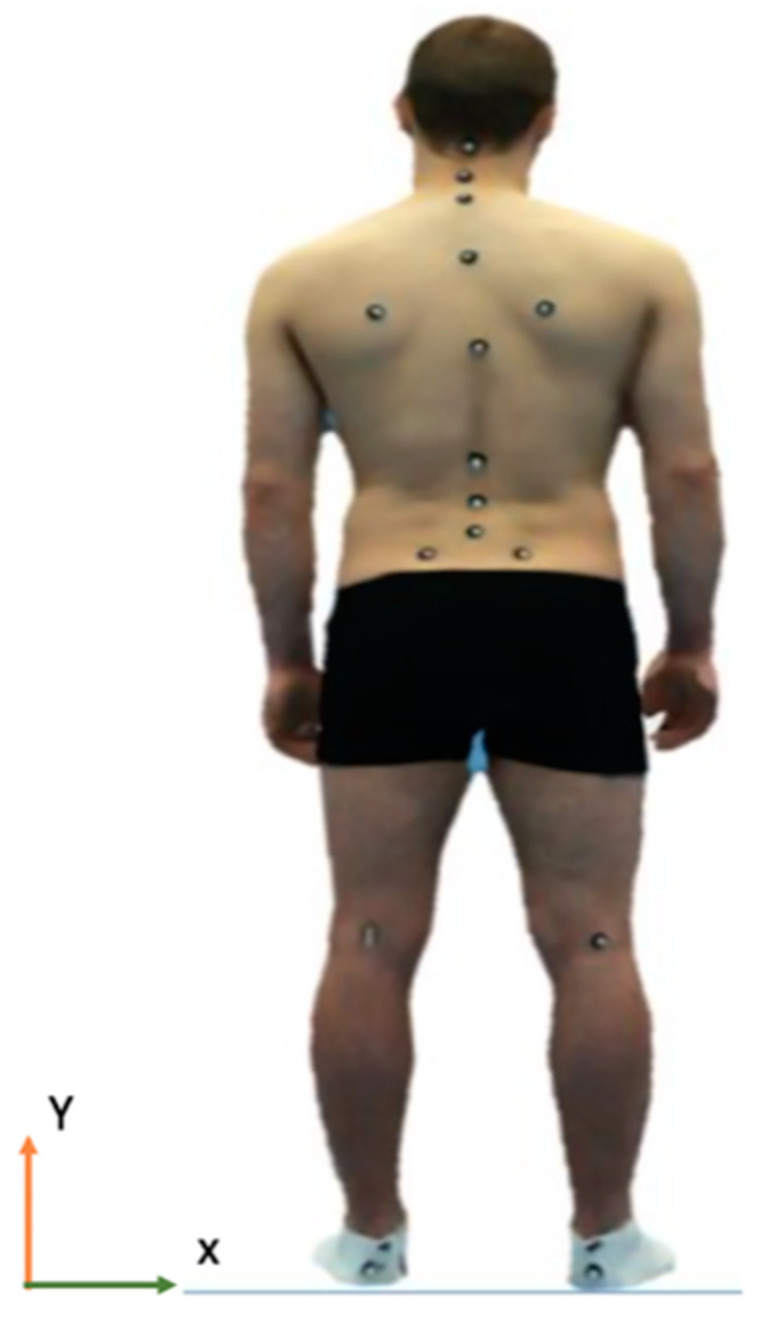
A participant standing with an anatomical marker set.

For each subject, five trained raters (two physiotherapists, two sports therapists, and a biomechanist) individually measured three trials of standing back and posture on two separate occasions to calculate both intra- and inter-rater reliability (Table 1). Each rater completed the palpation of bony landmarks and placed nineteen 10mm spherical reflective markers on the anatomical landmarks (Figure 1).

**Figure 2:**
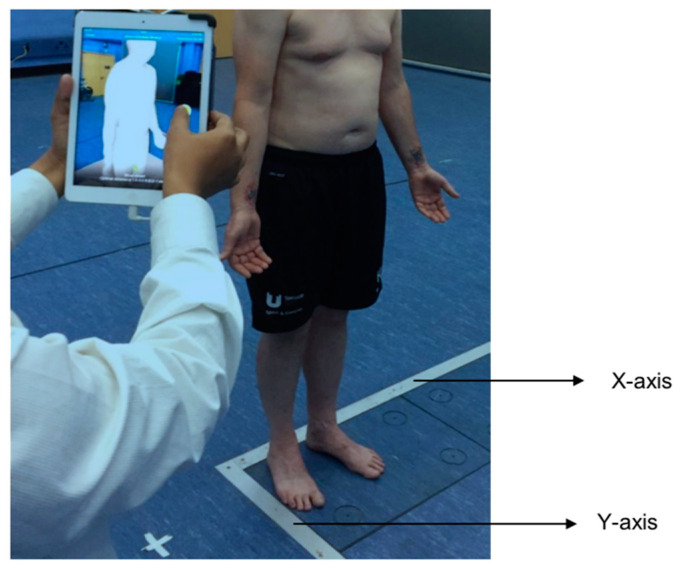
A participant standing with feet placement towards the X and Y axes.

Anatomical reference points, such as the midpoint of the patella and ankle joint line, were also used for angle calculation. Following the familiarization procedure, each participant’s standing 3D back shape and whole body were captured manually by the raters with the commercially available iPad camera and Structure Sensor^TM^ (Version 2018). A footplate was created with marks to standardize foot position, and a chart was placed on the wall in front of the subject with markers to focus on (Figure 2). Previous work has found improvements in repeated measurements with foot and vision standardization [14,15,16].

Participants were asked to look straight ahead and stand in a comfortable position. To avoid any bias in trial selection and obtain a better estimate of the raters’ true score, the mean of three trials for each rater was used to determine level of reliability. In order to capture the data, the rater walked around the subject, with the camera pointing towards the subject in a 360-degree circle at three different heights (participant’s shoulder, pelvic, and knee). The data acquisition followed a specific sequence, as explained above, and took 30 to 40 s per trial. Throughout data collection, the distance between the camera and the participant was between 0.5 to 1 m. In order to limit variability associated with participant positions and standardize the data collection process, two reference lines for foot placement were drawn on the floor at the X and Y axes.

All data captured through the 3D imaging MSTS were uploaded, realigned, and processed through the open-source software, Netfabb Basic^TM^. With the use of this software, nine postural variables and angular displacements, as described in Table 2, were individually measured by the raters.

## 3. Data Analysis

Intra-class correlation (ICC) was used to test the intra-rater and inter-rater reliability of posture and back shape variables, together with the standard error of measurement. Based on the thresholds provided by Portney and Watkins, (2000) [17], poor intra-class correlation coefficients are interpreted as fair or below fair if below 0.50, while acceptable ICCs are deemed reliable and valid if they are found to be moderate to good (0.50–0.75) or good to excellent (>0.75). To further examine the agreement between the trials and the raters for each posture variable, 90% confidence intervals (CIs) were used.

The interpretation of reliability data is more meaningful when ICC analysis is complemented with another test, the standard typical error (STE; the standard deviation of differences within a pair of raters or trials), which was performed [18,19]. As STE is a standard deviation, the usual scale of standardized effect sizes was halved to interpret STE magnitudes [20]. These thresholds are 0.1 for a small error, 0.3 for a moderate error, and 0.6 for a large error. Customized spreadsheets were used for all calculations of ICC and STE [21]. Quantitative data were analyzed with IBM Statistical Package for the Social Sciences (SPSS Version 23.0) software.

## 4. Results and Discussion

The objective and evidence-based evaluation of postural parameters are essential for healthcare practitioners to enable both the quantitative assessment of spinal conditions, as well as the longitudinal evaluation of clinical interventions. The study evaluates the use of a portable, 3D MSTS to quantify posture during standing. The results indicated good to excellent intra-rater and good to moderate inter-rater reliability for measuring 78% (7 out of 9) of postural variables with an ICC ranging from 0.70 to 0.98. The remaining 22% of variables (2 out of 9; lateral pelvic tilt and right frontal knee angle) showed moderate to low inter- and intra-rater reliability with ICCs ranging from 0.26 to 0.79 (Table 3, Table 4 and Table 5).

While the STE values in the current study had a wide range of scores with a large magnitude, it is important to acknowledge that the overall change in means between trials were very low (0.94–2.43° for intra-rater and 1.35–5.03° for inter-rater reliability). In biomechanical assessments, mean difference scores of 5 or less than 5 degrees are within the standard acceptable range of errors [22]. De Carvalho et al. [23] evaluated the intra- and inter-examiner reliability and reproducibility of goniometry in relation to photogrammetry of the hand, comparing the angles of thumb abduction, Proximal Inter Phalangeal (PIP) joint flexion of the second finger, and metacarpophalangeal (MCP) joint flexion of the fifth finger. The results of the study reveal that no significant differences were found between the groups for most of the measurements, and any difference in angle less than 5 degrees is acceptable in biomechanics studies.

An overall comparison of change in means and STEs does, however, show an apparent contradiction: the former (difference in means) indicates clinically consistent very small differences, whereas the latter (STE scores) varies considerably. The results of the current study were similar for both intra-rater as well as inter-rater reliability for most posture variables in the sagittal, frontal, and transverse planes.

### 4.1. Sagittal Plane Variables

The overall 3D MSTS demonstrated excellent to good reliability results for measuring External low back curvature, thoracic kyphosis, and external neck curvature.

#### 4.1.1. External Low Back Curvature (LBC)

In the current study the external neck curvature angle is formed by the intersection of lines drawn through the T12 spinous process to the apex of the lordosis (L3 vertebrae) and the apex through the S1 spinous process. The mean external neck curvature angle (27.37 + 5.4 degrees) presented in the study is similar to that from previous studies using the same calculation. Vialle et al. [24] reported that the LBC angle measured by the radiography method can widely vary between 14 degrees to 69 degrees. Similarly, De Oliveira Pezzan et al. [25] reported an LBC angle in an adolescent female (n = 50) normal healthy population as 40 ± 5.3 degrees when measured through the photographic method.

The measurement of LBC using the 3D MSTS demonstrated excellent intra-rater reliability (ICC value of 0.94) and good inter-rater reliability (ICC value of 0.79). The results of the current study are similar to previous studies using photography, radiography, and Moiré topography methods where a mean intra class correlation coefficient (ICC) > 0.98 was found [26,27,28,29,30].

Furthermore, in the current study, together with good reliability, the absolute changes in mean values across trials that achieved 90% confidence limits was as low as 1.45°. This is similar to the surface topography method Frerich et al. [30] demonstrated with a 2.1° difference between trials. The good intra-rater reliability (retest reproducibility) with small STE values for the measurement of LBC variables makes the MSTS an ideal tool to use within a clinical environment; one that is comparable to photogrammetry and radiography.

#### 4.1.2. Thoracic Kyphosis (TK)

The TK angle is formed by lines drawn through the upper end vertebrae of the curve (C7 spinous process) to the apex of the kyphosis and the apex through the lower end vertebrae of the curve (L1 spinous process). The mean TK angle (25.3 + 5.6 degrees) presented in the current study was similar or marginally lower than in previous studies. Gerald T, Michael J, and Thies (1980) [31] reported the normal TK angle to be 26.27 + 8.12 degrees in the age group 20–29 years and 29.04 + 7.93 in the age group 30–39 years. It is important to note that the variability in the TK angle is due to the different population groups and variations in the instruments, as well as in the measurement methods.

With regards to the reliability of measuring TK, the MSTS demonstrated excellent intra-rater reliability (ICC value of 0.87) and moderate inter-rater reliability (ICC value of 0.56). The results were similar to those of previous studies using the flexi-curve [32,33], photogrammetry [27,34,35], inclinometer [36,37,38], radiography [39], and Moiré topography [30,40] methods, with mean ICC > 0.83.

The absolute differences in mean values of TK were as low as 2.37 across trials with 90% confidence limits in the present study. The good intra-rater reliability (retest reproducibility) together with the moderate STE value for the measurement of TK variables makes the MSTS a suitable tool to use within the clinical environment, comparable to photogrammetry.

#### 4.1.3. External Neck Curvature (NC)

The NC angle is formed by the intersection of lines drawn through C2 and C4 spinous process and through C4 and C7 spinous process. The mean NC angle (31.12 + 7.33 degrees) presented in the current study was similar to previous studies. Harrison, Barry-Greb, and Wojtowicz; Grob, Frauenfelder, and Mannion; Da Motta, De Rezende Pratali, and De Oliveira; Hey et al. [41,42,43,44] reported that the mean cervical lordotic angle ranged from 25.2 to 39.2 degrees when measured using the radiography method. Further, Been, Shefi, and Soudack [45] identified no difference in the NC angle when comparing between genders. The non-symptomatic (n = 61) males presented with 39.2 + 11.5 degrees of NC and females (n = 60) with 36.7 + 9.5 degrees. Similarly, Abelin-Genevois et al. [46] reported normal NC angles in a paediatric population as 32.1 + 11.3 degrees. Therefore, there is no single universally applicable method for measuring the NC angle.

Within radiography, Ohara et al. [47] described several techniques to measure the NC angle in lateral radiographs; for example, an angle formed between C1 and C7 or C2 and C7 endplates. Similar to the present study, the angle formed between C2–C7 angle is the most widely reported method for measuring the NC angle in the sagittal plane [42,48,49,50]. Harrison et al. and Shin et al. [51,52] reported good intra-rater (ICC, 0.97) and inter-rater reliability (ICC, 0.95), with a smaller standard error of measurement when the NC angle was measured through lateral cervical radiographs. Furthermore, Raupp et al. [53] reported that the low-cost, easy-to-use flexi-curve method produced good to moderate intra-rater (ICC = 0.65; *p* = 0.001) and inter-rater (ICC = 0.679; *p* > 0.01) reliability in measuring the NC angle. These results are similar to those of the current study. The NC angle measured through the 3D MSTS demonstrated excellent intra-rater reliability (ICC = 0.92) and moderate inter-rater reliability (ICC = 0.63).

Overall, each method of evaluating the sagittal plane variable has its advantages and disadvantages, but the major problem is that it is difficult to compare measurements when performed by different methods and instruments. The main advantage of measurements using the MSTS is the lack of radiation, thus allowing for the frequent evaluation of spinal curves and monitoring of changes in the alignment of the spine in the sagittal plane.

### 4.2. Frontal Plane Variables

Despite good to excellent reliability for the majority of sagittal plane postural variables, moderate to low inter-rater and intra-rater reliability was found for two frontal plane variables (shoulder elevation and lateral pelvic tilt). Additionally, these variables had larger STE values with wider limits of agreement.

#### 4.2.1. Shoulder Elevation (SE)

The SE angle is formed by a line drawn between the left and right acromion process markers and the angle of this line to the horizontal. The mean SE angle (3.9 + 1.56 degrees; range between 1.74 and 7.04 degrees) presented in the study was marginally higher than those found in previous studies. Ferreira et al. [54] reported that the mean SE angle measured by the photographic method was 1.3 + 2.0 degrees (ranging from 3.5 to 7.0 degrees) in healthy young adults. The marginal variations of 2 degrees in the SE angle may be due to the different instruments used.

With regards to the reliability of measuring SE, the 3D MSTS demonstrated moderate intra-rater reliability (ICC value of 0.60) with low inter-rater reliability (ICC value of 0.26). In contrast, the photographic method for measuring the SE angle demonstrated high reliability (ICC = 0.89) [55,56]. Good to moderate intra-rater reliability (retest reproducibility) for measuring the SE angle makes the MSTS an excellent tool to evaluate this coronal plane variable in the clinical environment, one that is comparable to photogrammetry.

#### 4.2.2. Lateral Pelvic Tilt (LPT)

Most pelvic parameters described in the literature used radiographic [57], photographic [29,54], or goniometric [58] methods. The evaluation of pelvic tilt in the coronal plane used in the present study was based on the posterior views of the frontal plane of the 3D model. The LPT angle was calculated between the horizontal line and the line joining the two posterior superior iliac spines (PSIS). The mean LPT angle presented in the current study (3.91 + 1.62 degrees; minimum 2.05 and maximum 7.64 degrees) was marginally higher than those reported in previous studies. Pinto et al. [59] presented the normal lateral pelvic tilt measured by a 3D optoelectronic device as being −0.77 + 1.83 degrees in small sample size of n = 14 healthy young adults. Using photogrammetry methods, Fortin et al. and Ferreira et al. [29,54] reported the coronal pelvic angle as being −1.9 + 3.2 degrees and −0.9 + 2.2 degrees, respectively, in a large sample size (n = 115). Even though the MSTS estimated the mean LPT angle to be 2 degrees higher than previously published results, it is difficult to compare, as the measurement method used in the current study was based on surface topography data.

The current study results demonstrated moderate intra-rater reliability (ICC value of 0.73) and low inter-rater reliability (ICC value of 0.09) when the LPT angle was measured with the MSTS. The potential reasons for poor inter-rater reliability might have been attributed to variations in the experience of raters and their palpation skills.

#### 4.2.3. Frontal Knee Angle (FKA)

The mean FKA angle presented in the study (3.94 + 1.77 degrees on the left side and 4.75 + 2.88 degrees on the right side) are the first reported FKAs using a surface topography method. As there are not many posture studies that have reported the FKA, it is difficult to compare the results of the current study with previously published results.

The intra-rater reliability was similar to previous studies. Fortin et al. and Tomkinson and Shaw [29,60] demonstrated excellent reliability when the FKA was measured using photography at different times on the same day, where an ICC of >0.95 was found. Like other frontal plane variables, the current study found poor inter-rater reliability with an ICC = 0.10. However, Berryman et al. [61] suggest that amplitudes of curvature lower than two-degree variations remain less relevant within clinical practice.

### 4.3. Transverse Plane Variables

#### Scapular Prominence (SP)

The mean SP angle presented in the study was 30.2 + 5.57 degrees on the right side and 28.6 + 7.71 degrees on the left side. Furthermore, numerous methods have been used to evaluate scapular prominence, ranging from complex radiographic methods [62] to simple two-dimensional photographic [63,64] and three-dimensional surface-topographic methods [65,66,67,68]. Non-standardized procedures together with various definition of SP make it very difficult to compare the current results with existing literature.

Likewise, the study presented excellent intra-rater (ICC = 0.93 and 0.98) and moderate inter-rater (ICC = 0.75 and 0.67) reliability when SP was measured using the novel MSTS. Therefore, this highly reliable MSTS was useful to measure both sagittal and transverse plane postural variables within the clinical environment. This was found to be comparable to radiography or other complex surface topographic methods.

## 5. Limitations

There are a few limitations to this study. Firstly, although the author used only a small and homogenous population, the author believes that the results also apply to other clinical populations (subjects with spinal deformity) as well as spinal pain patients. The MSTS, together with the methodology, was successfully able to measure small angular differences between subjects, trials, and raters accurately. This is important for clinical use. Secondly, although the current study minimized the measurement error by setting up standard procedures and training the raters, the results need to be interpreted with caution, as this study could not quantify any influence of postural sway on the results. Further large research studies are needed to evaluate this.

## 6. Further System Development and Research

Developing a tool with automatic recognition of markers as well as the calculation of automatic angles may potentially decrease measurement error and further reduce the time required to collect and analyze posture data. Future development of an automated, bespoke 3D posture mobile application could further benefit clinicians by decreasing the time required for data capture and help improve the reliability of measurements, as well as evidence-based clinical practice. The feasibility of using more than one structure sensor together with synchronized data capture will further help by not only decreasing the duration but will also improve the quality of the data for both screening patients as well as for the assessment of patients with back pain or different types of spinal disorders.

Since the MSTS used in this study was found to have moderate to good reliability for the measurement of sagittal and transverse plane postural variables, several possible future studies can be identified. As the posture and back shape variables differ in populations with different ages, genders, and ethnicities, studies with a larger and wider sample size on healthy participants could provide a normative database for a wide range of the population. Additionally, the MSTS has the potential to measure more posture variables, for example, forward head posture.

## 7. Conclusions

The current study has examined the inter-rater and intra-rater reliability of a mobile application-based 3D modelling method for the objective quantification of clinically identified postural alignment. The variables of the neck, trunk, and lower limb in standing were measured. This is the first study to evaluate the inter-rater and intra-rater reliability of the MSTS system. The results indicated good to excellent intra-rater and good to moderate inter-rater reliability for measuring 78% (7 out of 9) of postural variables. The remaining 22% of variables (2 out of 9; lateral pelvic tilt and right frontal knee angle) showed moderate to low inter- and intra-rater reliability. These reliability results provide a base for future studies. This device has the potential to be used as a complementary tool alongside subjective assessment for patients with a wide variety of spinal pathologies.

## Figures and Tables

**Table 1:** Descriptive statistics of both participants and raters.

	*n*	Gender	Age (Years) Mean (SD)	Height (cm) Mean (SD)	Mass (kg) Mean (SD)	Profession	Years of Experience Mean (SD)
Participants	16	Male	25 (5.6)	172 (5.3)	69 (8.6)	-	-
Raters	5	Male (4)	36 (7.6)	-	-	Physiotherapist (2) Sports Therapist (2) Bio-mechanist (1)	10 (7.0) 8.5 (4.9) 2
		Female (1)	32	-	-		

**Table 2:** Definitions of the nine postural angles measured by the raters.

Angle	Description	Picture
External Low Back Curvature	The angle formed by lines drawn through upper end vertebrae of the curve to the apex of the lordosis (T12) and the apex through S1	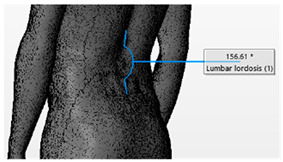
Thoracic Kyphosis Angle	The angle formed by lines drawn through the upper end-vertebra of the curve (T1) to the apex of the kyphosis and the apex through the lower end-vertebra of the curve (T12)	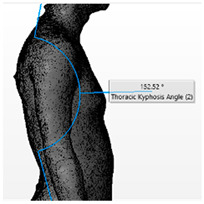
External Cervical Angle	The angle formed by lines drawn through C2 and C4 and through C4 and T1	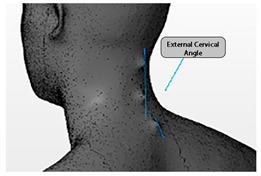
Shoulder Elevation in Angles	The angle formed by a line drawn between the left and right acromion process markers and the angle of this line to the horizontal	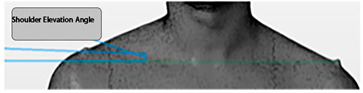
Lateral Pelvic Tilt in Angles	The angle formed by the horizontal and by the line joining the two PSIS	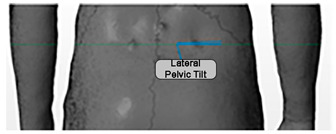
Frontal Knee Angle (Right) Frontal Knee Angle (Left)	The angle of intersection from a line drawn between the ASIS and the mid-pole of the patella, and a second line drawn between the mid-pole of the patella and the talus	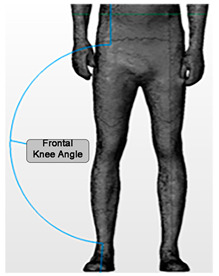
Scapular Prominence (Right) Scapular Prominence (Left)	The angle formed by lines drawn through T8 vertebrae of the curve to the apex of the scapula prominence and the lower end angle of the curve (midline to axilla)	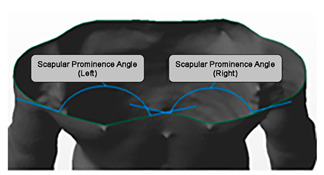

**Table 3:** Mean, standard deviation (SD), and minimum and maximum values of postural variables in all planes.

	Variables (In Degrees)	Trial 1		Trial 2		Trial 3		Overall Mean of All Three Trials	SD of All Three Trials	Min	Max
		Mean	SD	Mean	SD	Mean	SD				
Sagittal Plane Variable	External low back curvature	27.3	5.7	27.4	5.9	27.4	5.4	27.37	5.41	18.66	34.92
	Thoracic kyphosis angle	25.3	5.9	26.6	5.8	24.3	6.6	25.36	5.6	13.55	34.08
	External neck curvatures	30.6	7.5	31.9	7.1	30.9	8.8	31.12	7.33	22.49	48.33
Frontal Plane Variable	Shoulder elevation in angles	3.9	2.2	3.9	1.8	4.2	1.6	3.99	1.56	1.71	7.04
	Lateral pelvic tilt in angles	3.6	2.4	3.9	1.9	4.3	1.3	3.91	1.62	2.05	7.64
	Frontal knee angle (Right)	4.7	3.0	4.9	3.4	4.6	3.0	4.75	2.88	0.96	9.12
	Frontal knee angle (Left)	3.8	2.0	4.0	2.1	3.9	1.8	3.94	1.77	1.23	6.72
Transverse Plane Variable	Scapular prominence (right)	30.2	6.2	30	5.2	30.6	6.7	30.27	5.57	21.14	39.73
	Scapular prominence (left)	27.9	8.7	28.9	8.1	28.9	7.9	28.6	7.71	16.12	40.68

**Table 4:** Intra-rater reliability of posture variables measured using the MSTS.

	Variables	Overall Change in Mean Difference between Trials (in Degrees); (±90% CI)	ICC R Value; (±90% CI)	Standardized Typical Error
Sagittal Plane Variables	External low back curvature	1.45 (1.18 to 1.94)	0.94 (0.88 to 0.97)	0.26 (Small)
	Thoracic kyphosis angle	2.37 (1.93 to 3.16)	0.87 (0.74 to 0.94)	0.39 (Moderate)
	External neck curvature	2.43 (1.98 to 3.24)	0.92 (0.84 to 0.96)	0.30 (Moderate)
Frontal Plane Variables	Shoulder elevation in angles	1.21 (0.94 to 1.78)	0.60 (0.24 to 0.84)	0.67 (Large)
	Lateral pelvic tilt in angles	0.95 (0.73 to 1.38)	0.73 (0.44 to 0.90)	0.57 (Moderate)
	Frontal knee angle (right)	0.94 (0.72 to 1.37)	0.93 (0.84 to 0.98)	0.30 (Moderate)
	Frontal knee angle (left)	0.79 (0.61 to 1.16)	0.87 (0.70 to 0.96)	0.40 (Moderate)
Transverse Plane Variables	Scapular prominence (right)	1.85 (1.42 to 2.70)	0.93 (0.83 to 0.98)	0.31 (Moderate)
	Scapular prominence (left)	1.37 (1.06 to 2.00)	0.98 (0.95 to 0.99)	0.17 (Small)

**Table 5:** Inter-rater reliability of posture variables measured using the MSTS.

	Variables	Overall Change in Mean Difference between Raters (in Degrees); (±90% CI)	ICC R Value; (±90% CI)	Standardized Typical Error
Sagittal Plane Variables	External low back curvature	2.80 (2.40 to 3.48)	0.79 (0.65 to 0.90)	0.48 (Moderate)
	Thoracic Kyphosis Angle	3.89 (3.33 to 4.83)	0.56 (0.36 to 0.75)	0.69 (Large)
	External neck curvature	5.03 (4.30 to 6.24)	0.63 (0.44 to 0.80)	0.63 (Large)
Frontal Plane Variables	Shoulder Elevation in Angles	1.36 (1.17 to 1.69)	0.26 (0.07–0.52)	0.87 (Large)
	Lateral Pelvic Tilt in Angles	1.35 (1.15 to 1.67)	0.09 (0.07–0.34)	0.96 (Large)
	Frontal Knee Angle (Right)	2.10 (1.79 to 2.60)	0.40 (0.20–0.64)	0.70 (Large)
	Frontal Knee Angle (Left)	2.14 (1.83 to 2.66)	0.10 (−0.06–0.35)	0.89 (Large)
Transverse Plane Variables	Scapular prominence (Right)	3.70 (3.16 to 4.59)	0.75 (0.60–0.87)	0.53 (Moderate)
	Scapular prominence (Left)	4.67 (4.00 to 5.80)	0.67 (0.49–0.82)	0.60 (Large)

## Data Availability

The data presented in this study are available on request from the corresponding author.
